# Blindfolding during wakefulness causes decrease in sleep slow wave activity

**DOI:** 10.14814/phy2.13239

**Published:** 2017-04-13

**Authors:** Eva Magdalena Korf, Matthias Mölle, Jan Born, Hong‐Viet V. Ngo

**Affiliations:** ^1^ Department of NeuroendocrinologyUniversity of LübeckLübeckGermany; ^2^ Center of BrainBehavior and MetabolismUniversity of LübeckLübeckGermany; ^3^ Institute for Medical Psychology and Behavioral NeurobiologyUniversity of TübingenTübingenGermany; ^4^ School of PsychologyUniversity of BirminghamBirminghamUnited Kingdom

**Keywords:** Humans, slow wave sleep, synaptic plasticity

## Abstract

Slow wave activity (SWA, 0.5–4 Hz) represents the predominant EEG oscillatory activity during slow wave sleep (SWS). Its amplitude is considered in part a reflection of synaptic potentiation in cortical networks due to encoding of information during prior waking, with higher amplitude indicating stronger potentiation. Previous studies showed that increasing and diminishing specific motor behaviors produced corresponding changes in SWA in the respective motor cortical areas during subsequent SWS. Here, we tested whether this relationship can be generalized to the visual system, that is, whether diminishing encoding of visual information likewise leads to a localized decrease in SWA over the visual cortex. Experiments were performed in healthy men whose eyes on two different days were or were not covered for 10.5 h before bedtime. The subject's EEG was recorded during sleep and, after sleep, visual evoked potentials (VEPs) were recorded. SWA during nonrapid eye movement sleep (NonREM sleep) was lower after blindfolding than after eyes open (*P* < 0.01). The decrease in SWA that was most consistent during the first 20 min of NonREM sleep, did not remain restricted to visual cortex regions, with changes over frontal and parietal cortical regions being even more pronounced. In the morning after sleep, the N75‐P100 peak‐to‐peak‐amplitude of the VEP was significantly diminished in the blindfolded condition. Our findings confirm a link between reduced wake encoding and diminished SWA during ensuing NonREM sleep, although this link appears not to be restricted to sensory cortical areas.

## Introduction

Slow wave sleep (SWS) which in humans occurs mostly during the early night, is characterized by predominant slow wave activity (SWA). Slow waves are EEG oscillations with a frequency of <4 Hz that are considered a correlate of the slow oscillation resulting from synchronized neuronal depolarization during up‐states and synchronized neuronal hyperpolarization during down states (Steriade et al. 1993). They have been shown to play an important role in the consolidation especially of declarative memories (Mölle and Born 2011; Rasch and Born 2013), as well as in the homeostatic regulation of synaptic potentiation in cortical networks (Tononi and Cirelli 2003, 2006, 2014).

Based mainly on computational models it has been proposed that increased SWA is an electrophysiological correlate of enhanced synaptic potentiation in cortical networks, inasmuch as a generally increased synaptic strength produces stronger synchronization of cortical neuronal activity and, thus, increased high amplitude SWA (Esser et al. 2007; Olcese et al. 2010). Consistent with this concept SWA is subjected to homeostatic regulation with, increased SWA after long periods of wakefulness and decreased SWA after sleep (Borbély and Achermann 2000). Furthermore, after learning a visuomotor task SWA during subsequent sleep increased over the cortical regions mainly involved in the task, that is regions of the right parietal lobe encompassing Brodman areas 40 and 7 (Huber et al. 2004). The local up‐regulation in post‐learning SWA was associated with an overnight improvement in task performance. On the other hand, short‐term arm immobilization locally reduced SWA over sensorimotor areas, produced an overnight impairment in arm motor performance, and also decreased motor potentials evoked by transcranial magnetic stimulation (MEPs) as well as somatosensory evoked potentials (SEPs), both recorded prior to sleep (Huber et al. 2006). The decrease in MEP and SEP responses after arm immobilization was interpreted as another reflection of reduced synaptic potentiation in respective motor and sensorimotor networks and, consequently, of reduced synchronization of responses evoked in these networks (Iwasaki et al. 2004). Collectively, these and a number of further studies indicate that SWA is increased after wake conditions supposedly increasing synaptic potentiation in specific networks, for example, by intensive training on certain tasks or specific experiences, whereas SWA is decreased after conditions supposedly preventing such net synaptic potentiation precluding specific stimulation and experiences.

In order to investigate whether these findings in the sensorimotor domain can be generalized to purely sensory systems we tested whether eye blindfolding, assumed to prevent synaptic potentiation in visual cortical areas, produces a comparable decrease in SWA specifically over occipital cortical areas, as has been, for example, observed following arm immobilization over the motor cortex. To this end we performed experiments in fifteen healthy men whose eyes were either covered for 10.5 h before bedtime or not. Besides sleep SWA, in the morning after sleep visual evoked potential responses were recorded, as another indicator of network synaptic potentiation and synchrony.

## Materials and Methods

### Subjects and procedures

Fifteen healthy, nonsmoking men (mean age 21.33 ± 0.73 years) with regular sleep‐wake rhythm during a period of 6 weeks before the experiments participated in the study. All subjects were informed about the experimental protocol and gave written informed consent prior to participation. The study was approved by the local ethics committee of the University of Lübeck.

After an adaption night, each subject participated in a “blindfolded” and an “eyes‐open” condition, with both conditions separated by at least 1 week. The order of the conditions was randomized across subjects. For blindfolding the participant's eyes were covered with orthoptic patches and an additional mask between 12.30 p.m., when the experiment started, and 7.00 a.m. the next day, when subjects were awakened. In both conditions, subjects were kept alert on a standardized low activity level during daytime (12.30 p.m. to 11.00 p.m.). Thus, they listened to a specific audio book in the blindfolded condition and read the corresponding book (but another passage) in the eyes‐open condition; they performed on corresponding auditory and visual versions of a computer game, and in both conditions listened to music and exercised on a bicycle ergometer. Following awakening, the mask and the orthoptic patches were removed and VEPs were registered. Blood was sampled hourly via an intravenous forearm catheter in a subsample of six subjects, for the determination of cortisol and melatonin levels as indicators of the circadian rhythm. During bedtime, blood samples were taken via a long thin tube through the wall from a neighboring room, without disturbing the subject's sleep. In both conditions, subjects estimated their subjective tiredness and their mood by answering standardized questionnaires at the beginning of the experiment and before bedtime.

### Recordings and analysis of sleep EEG

**Figure 1:**
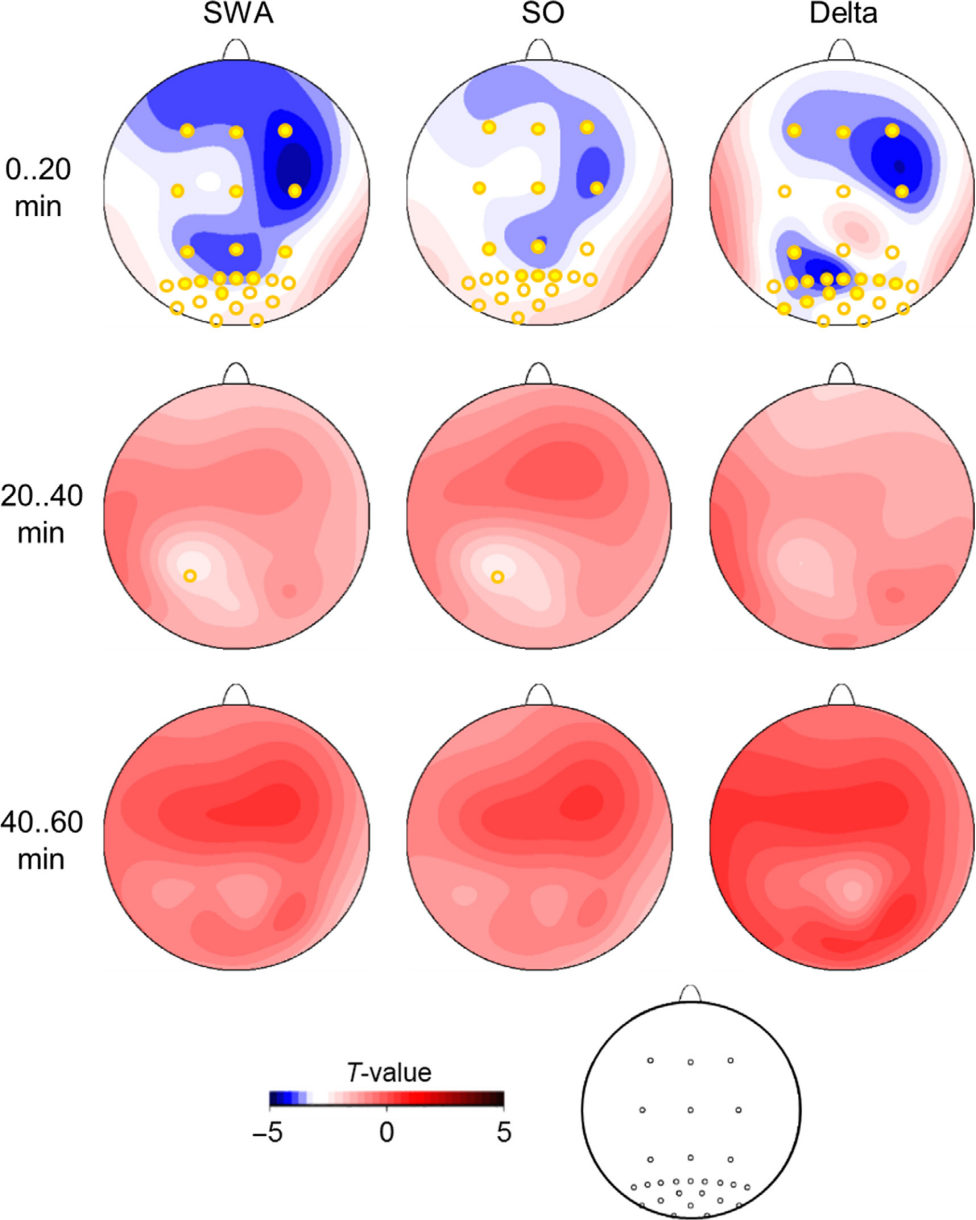
Difference in EEG power during the first three 20‐min intervals of NonREM sleep for slow wave activity (SWA, 0.5–4 Hz), slow oscillation (0.5–1.0 Hz), delta (1–4 Hz) frequency bands (*n* = 13). Differences are indicated by statistical *t*‐values with negative values indicating lower power for the blindfolded than the eyes‐open condition. Significant differences at specific electrode locations are indicated by filled yellow circles (*P* < 0.01) and unfilled yellow circles (*P* < 0.05). A schema of electrode positions is shown at the bottom.

The EEG was recorded during sleep from 27 sites (using a Neurofax EEG‐9200 amplifier, Nihon Kohden, Tokyo, Japan). EEG signals were sampled at a frequency of 500 Hz filtered between 0.16 and 70 Hz. Electrodes were placed according to a modified 10–20 system covering visual cortical areas at an increased spatial resolution (F3, Fz, F4, C3, Cz, C4, P3, Pz, P4, O1, Oz, O2, PO7, PO5, PO3, PO1, POz, PO2, PO4, PO6, PO8, POO9 h, POO1, POO2, POO10 h, OI1 h, OI2 h, see Fig. 1), referenced to linked electrodes attached to the mastoids. Horizontal and vertical eye movements as well as submental electromyogram were recorded for standard polysomnography.

The analysis of sleep EEG data comprised two steps. First, sleep structure was determined visually according to standard polysomnographic criteria (Berry et al. 2015), based on EEG signals from C3 and C4. Stage N2 sleep corresponds to light nonrapid eye movement (NonREM) sleep and stages N3 corresponds to SWS. Movement times and epochs with artifacts were excluded from further analysis. After sleep stage scoring, EEG data were processed using Spike2 software (Cambridge Electronic Design Limited, Cambridge, UK). EEG power was analysed for all NonREM sleep epochs using Fast Fourier Transformation (FFT). FFT was calculated in blocks of 8192 data points, corresponding to two succeeding blocks for every 30‐sec epoch scored as sleep stage N2 or N3. Average power was then determined for the first three 20‐min intervals of NonREM sleep and for NonREM sleep of the remaining night, for five frequency bands of interest: that is, the slow‐wave band (0.5–4 Hz), the slow‐oscillation band (0.5–1 Hz), the delta band (1–4 Hz), the slow‐spindle band (9–12 Hz) and the fast‐spindle band (12–15 Hz).

Additionally, discrete slow oscillations (SOs) were identified separately in all channels for the same first three 20‐min intervals of NonREM sleep as used for power analysis. SO detection was based essentially on a standard algorithm described elsewhere in detail (Mölle et al. 2002). Briefly, in a first step, the EEG was low‐pass filtered at 30 Hz and down‐sampled to 100 Hz. For the identification of large SOs, a low‐pass filter of 3.5 Hz was applied to the EEG, and time points of positive to negative zero crossings were computed in the resulting signal. In all intervals of positive to negative zero crossings with a length of 0.8–2 sec (corresponding to 0.5–1.25 Hz) the lowest and highest value between every 2 of these time points were detected (i.e., one negative and one positive peak between 2 succeeding positive to negative zero crossings). For a subject a SOs was identified if two individual threshold criteria were met, one for the negative peak amplitude and the other for the negative‐to‐positive amplitude difference. To define these thresholds, for the individual's eyes‐open condition the amplitude values of the negative and positive peaks were averaged across all channels and multiplied by 1.5 (which resulted in average voltage criteria of ‐66.3 ± 3.8 *μ*V and 125.3 ± 7.1 *μ*V, for the negative peak amplitude and for the negative‐to‐positive amplitude difference, respectively). For both conditions of a subject only those intervals of 0.8–2 sec length whose amplitude values exceeded the two thresholds were marked as SO events. For every channel and each of the three 20‐min intervals four features of the SO events were calculated: density (number of SO events per 30 sec), mean negative peak amplitude, mean negative to positive peak amplitude and mean slope (negative peak to next zero crossing).

### VEP recordings and analysis

VEPs were recorded ~15 min after awakening according to standards of the ophthalmologic society (Odom et al. 2004, 2016; Drislane 2007), using the same Neurofax EEG‐9200 amplifier, amplifier settings and electrode montage as for the EEG sleep recordings, that is, 27 EEG channel referenced to linked mastoid and with a ground electrode placed at the forehead. Checkerboard reversals were presented at a rate of 1 Hz in 6 blocks of 100 stimuli differing in black/white contrasts with two blocks of 100%, 85% and 70% of full brightness. Due to a technical confound that led to missing markers for the checkboard reversals of 100% and 70% contrast, this report is restricted to the medium contrast. Checkerboards consisted of 192 squares, each check subtended 1.1 degrees of visual angle. During recordings, the subjects sat relaxed in a reclining chair in a darkened room at a distance of ~1 m in front of the stimulus screen (15″ monitor color cathode ray tube, MultiScan 5FGe, NEC, Munich, Germany), that subtended a horizontal visual angle of 13 degrees. They were requested to fixate on a centrally located cross and to avoid eye movements or blinking during the stimulus presentation. To maintain the subject's attention, after every 10 reversal stimuli, during a 12‐sec break a black screen was presented with the fixation point in the middle but changing its color every 3.5–5.0 sec. Subjects were asked to press a button as fast as possible whenever they detected a color change.

VEPs were analyzed using the BrainVision Analyzer software (Brain Products GmbH, Gilching, Germany). For all checkerboard reversals of the medium contrast the EEG (low pass filtered at 35 Hz, 48 dB/oct roll‐off) was subdivided into 450–ms epochs including a 25–msec prestimulus baseline. Blink artifacts were removed from the data using an independent component analysis (ICA) ‐based method, as implemented in the BrainVision Analyzer software. Subsequently, epochs contaminated by movement artifacts exceeding a threshold of ±75 *μ*V were entirely removed, yielding 157.9 ± 11.8 and 151.6 ± 12.2 trials for the eyes‐open and blindfolded condition, respectively (*P* = 0.457, students' *t*‐Test). Before averaging, all epochs were baseline corrected by subtracting the average potential during the ‐25–0 msec prestimulus onset interval. A short baseline was chosen to avoid overlap with late components from foregoing responses (Using a longer 100‐msec prestimulus onset baseline did not essentially change the results). In the averaged signal, the following components were identified: P50 was defined as the most positive peak between 40 and 60 msec, N75 as the most negative peak between 50 and 90 msec, P100 as the most positive peak between 80 and 120 msec and N145 as the most negative peak between 130 and 170 msec. Peak latencies, peak amplitudes of the P50, N75, P100 and N145 components as well as the peak‐to‐peak amplitudes P50‐N75, N75‐P100 and P100‐N145 were calculated in all channels.

### Analysis of hormones and subjective measures

Cortisol levels were determined using a commercial chemiluminescent enzyme immunoassay (Cortisol‐Immulite 1000, Siemens Healthcare Diagnostics GmbH, Eschborn, Germany, sensitivity 0.2 *μ*g/dl, intra‐assay coefficient of variation 5.8–8.8%) and melatonin levels by means of a radio immunoassay (Melatonin direct RIA, IBL International, Hamburg, Germany, sensitivity 0.9 pg/mL, intra‐assay coefficient of variation 3.9–6.9%). All samples from an individual were assessed in the same assay.

For estimation of subjective tiredness, we used the Stanford Sleepiness Scale which consists of seven shorthand descriptions of alertness or sleepiness, respectively. The subject is asked to choose the one best fitting his actual condition (Hoddes et al. 1973). The subject's mood was assessed using the “Mehrdimensionale Befindlichkeitsfragebogen – Kurzform A” (Steyer et al. 1997) in which the subject is asked to rate on five‐point rating scales, for 12 different adjectives, to what extent an adjective fits his current mood state.

### Statistical analyses

For the sleep EEG and VEP analyses data from two subjects each were excluded, because of poor quality of sleep, EEG signal, or due to movement artifacts. Statistical analyses of EEG power and SO parameters generally relied on analyses of variance (ANOVA) including a repeated measures factor Blindfolding (blindfolded vs. eyes‐open conditions), Analyses of SOs included an additional repeated measures factor Topography (27 EEG channels). First, effects were tested for the total night then, based on previous findings indicating that effects after deprivation form sensory motor inputs are restricted to the very early night (Huber et al. 2006), we tested for effects within the first 20 min of NonREM sleep, as well as for effects on the first 60 min. The latter analyses included an additional repeated measures factor Time interval (1st, 2nd, 3rd 20‐min interval within the initial 60 min of NonREM sleep). Posthoc pairwise testing was applied to specify significant ANOVA main and interaction effects. For weaker effects (0.05 > *P* > 0.025) robustness of the effects was additionally examined using permutation tests (which were cluster‐based for Topography effects) and involved 2000 permutations (Huber et al. 2004; Groppe et al. 2011).

Analyses of VEPs were likewise based on an initial ANOVA including factors Blindfolding and Topography. Based on previous work indicating the localized nature of the VEP components of interest (e.g., Odom et al. 2004, 2016) the Topography factor was restricted to the 17 electrode sites covering the parietal and occipital cortical areas (P3, Pz, P4, O1, Oz, O2, PO7, PO5, PO3, PO1, POz, PO2, PO4, PO6, PO8, POO1 and POO2). Analyses of VEPs were additionally conducted after re‐referencing the signal to Fz. As these analyses revealed essentially the same results, this report is restricted to the analysis of the original VEP signal referenced to linked mastoid references. Sleep stages, hormonal and subjective data were analyzed using paired *t*‐tests. A *P* < 0.05 was considered significant.

## Results

### Sleep architecture

Subjects displayed normal sleep patterns in both conditions. Across the whole night, the amount of SWS was higher in the blindfolded than the eyes‐open condition (means ± SEM: 80.8 ± 8.6 min vs. 69.2 ± 7.9 min; *P* = 0.03; Table 1). The amount of N2 sleep was lower after blindfolding than eyes‐open (235.1 ± 7.8 min vs. 261.7 ± 9.1 min; *P* = 0.012). REM sleep as well as latency of sleep or sleep stages were not significantly affected by blindfolding.

**Table 1:** Sleep architecture for the entire night and the 1st, 2nd, and 3rd 20 min of NonREM sleep

Parameter (in min)		Eyes‐open		Blindfolded		*t*‐test
		Mean	SEM	Mean	SEM	
Sleep latency		24.8	4.9	17.4	3.0	0.160
SWS latency		24.1	7.5	18.5	1.8	0.491
REM latency		96.0	78.9	11.2	9.8	0.150
Waking after sleep onset		14.2	2.8	23.8	7.8	0.241
N1		35.1	4.7	33.2	4.8	0.606
N2		261.7	9.1	235.2	7.8	**0.012**
SWS		69.2	7.9	80.8	8.6	**0.030**
NonREM sleep		330.9	10.0	316.0	7.6	0.187
REM sleep		86.8	5.5	94.8	6.0	0.074
Movement time		2.8	0.7	2.5	0.5	0.700
1st 20 min	N2	13.1	1.1	15.1	1.0	**0.035**
	SWS	6.9	1.1	4.9	1.0	
2nd 20 min	N2	7.0	1.9	6.5	1.5	0.733
	SWS	13.0	1.9	13.5	1.5	
3rd 20 min	N2	10.9	1.6	9.7	1.8	0.283
	SWS	9.1	1.6	10.3	1.8	

SWS, slow wave sleep (N3), significances in bold, *n* = 13.

If only the first 20 min of NonREM sleep were analyzed, subjects after blindfolding, conversely, spent less time in SWS than after eyes‐open (4.9 ± 1.0 min vs. 6.9 ± 1.1 min; *P* = 0.035). but more time in N2 sleep after blindfolding (15.1 ± 1.0 min vs. 13.1 ± 1.1 min; *P* = 0.035, Table 1). There were no differences between conditions in the second and third 20 min periods of NonREM sleep. Analysis of the remaining night revealed the same pattern as the whole night analysis with more SWS (51.3 ± 7.5 min vs. 39.4 ± 5.8 min; *P* = 0.017) and less N2 sleep (200.8 ± 7.7 min vs. 228.6 ± 9.9 min; *P* = 0.016) after blindfolding than eyes‐open (Table 1).

### EEG Power

Average power spectra across the initial 20 min of NonREM sleep indicated the expected prevalence of SWA over anterior cortical regions. SWA during this time was significantly lower in the blindfolded than eyes‐open condition (*F*_(1,12)_ = 11.118; *P* = 0.006, Fig. 1). ANOVA on the first 60 min of NonREM sleep also revealed a main effect of *Blindfolding* (*F*_(1,12) _= 5.333; *P* = 0.04) but, SWA did not differ between conditions when tested separately for the second and third 20 min of NonREM sleep, or in tests for the remaining night (*P* > 0.164 for all relevant comparisons). Effects of blindfolding on SWA in these analyses did not show any local focus (*P* > 0.069, for respective *Blindfolding* x *Topography* interactions).

The effects of blindfolding on SWA were likewise revealed in both sub‐bands, that is, the slow oscillation band (0.5–1 Hz) and the delta band (1–4 Hz). After blindfolding slow oscillation power was distinctly reduced in the first 20 min of NonREM sleep (*F*_(1,12) _= 7.593; *P* = 0.017; Fig. 1), and ANOVA across the first 60 minutes of NonREM sleep still revealed a trend towards decreased SWA after blindfolding (*F*_(1,12) _= 4.104; *P* = 0.066, for *Blindfolding* main effect). Again the effects showed no specific topography (*P* > 0.573, for respective *Blindfolding* × *Topography* interactions). Similarly, power in the delta band tended to be lower after blindfolding than eyes‐open during the initial 20 min of NonREM sleep (*F*_(1,12)_ = 15.696; *P* = 0.002), with the ANOVA on the first 60‐min period of NonREM sleep revealing a trend towards an effect in the same direction (*F*_(1,12) _= 4.464; *P* = 0.056). In contrast to SWA and slow oscillatory power, the decrease in delta activity during the first 20 min of NonREM sleep showed topographical specificity (*F*_(26,312) _= 7.271; *P* = 0.002; for *Blindfolding* × *Topography*), with most robust decreases after blindfolding observed over right fronto‐central and left parieto‐occipital areas, although the effect was also significant over other regions including the occipital visual cortex regions (Fig. 1). There were no differences between blindfolding and eyes‐open conditions in the slow oscillation, or delta band in the second or third 20 min of NonREM sleep or in the remaining night (*P* > 0.185, for all comparisons).

**Figure 2:**
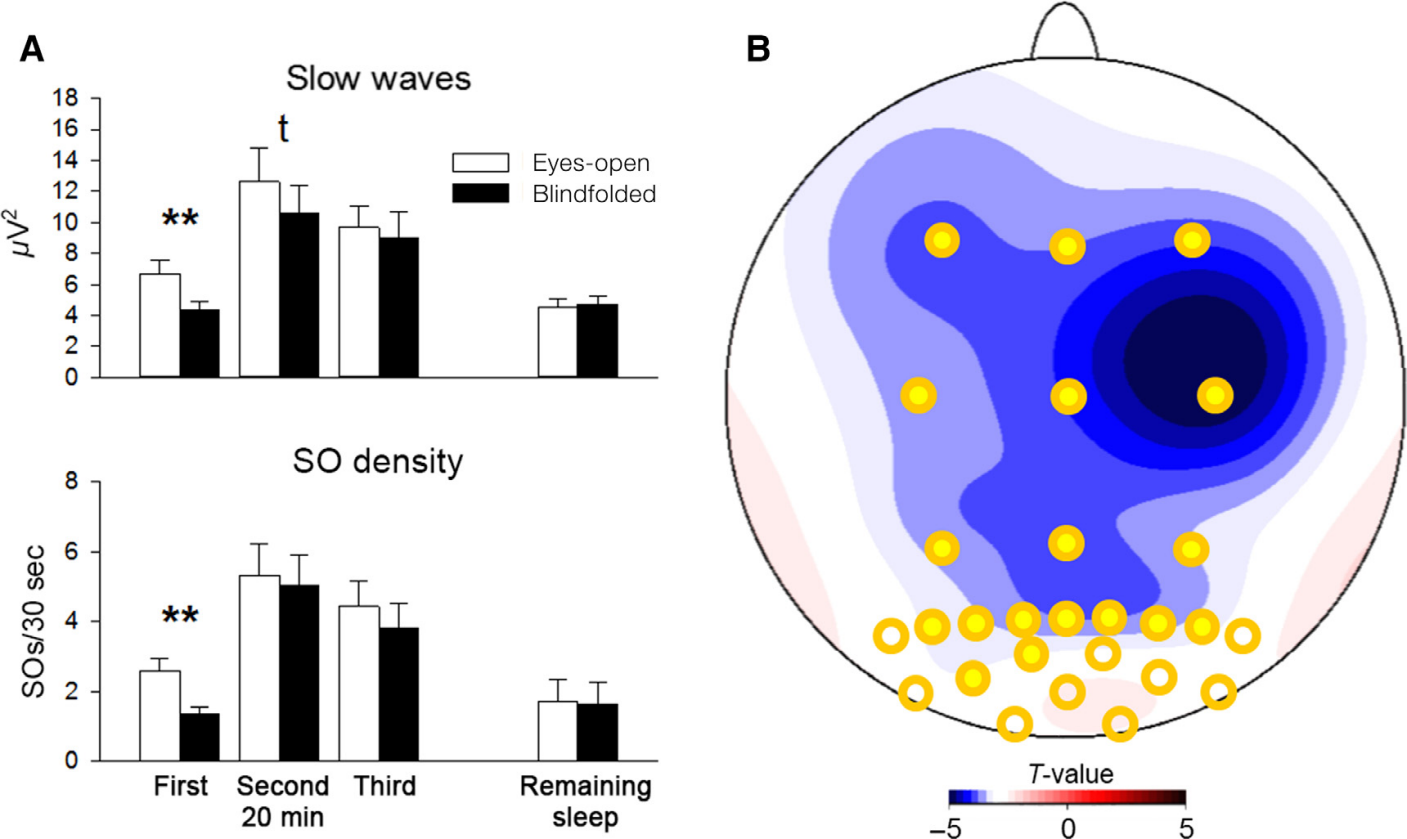
Changes in SO density and SWA after blindfolding (*n* = 13). (A) Difference in SO density during the first 20‐min interval of NonREM sleep. Differences are indicated by statistical *t*‐values with negative values indicating lower density for the blindfolded than the eyes‐open condition. Significant differences at specific electrode locations are indicated by filled yellow circles (*P* < 0.01) and unfilled yellow circles (*P* < 0.05). (B) Time course of changes in SWA (0.5–4 Hz) and SO density during NonREM sleep of the first three 20‐min intervals and the remaining night. Means ± SEM from parieto‐occipital electrode sites (for illustrative purposes pooled across PO1, POz, PO2) are shown. ^t^ *P* < 0.1, **P < 0.01. SWA, Slow wave activity.

Identification of discrete slow oscillations (SOs) corroborated results of the SWA analyses. SO density was strikingly lower after blindfolding than eyes‐open during the first 20 min of NonREM sleep (*F*_(1,12) _= 12.961; *P* = 0.004, Fig. 2A), and still tended to be lower in an ANOVA across the first 60 min of NonREM sleep (*F*_(1,12) _= 4.207; *P* = 0.063, for *Blindfolding* main effect, Fig. 2A). SO density after blindfolding was most consistently decreased over right fronto‐central areas (*F*_(26,312) _= 4.983; *P* = 0.007; for *Blindfolding* × *Topography*, Fig. 2B). There was no effect of blindfolding on any other of the SO parameters (including peak‐to‐peak amplitude, negative peak amplitude, and SO slope) or during later NonREM sleep periods of the night. Also, blindfolding did not induce any significant changes in slow (9–12 Hz) or fast (12–15 Hz) spindle activity (all *P* > 0.064).

### VEP responses

**Figure 3:**
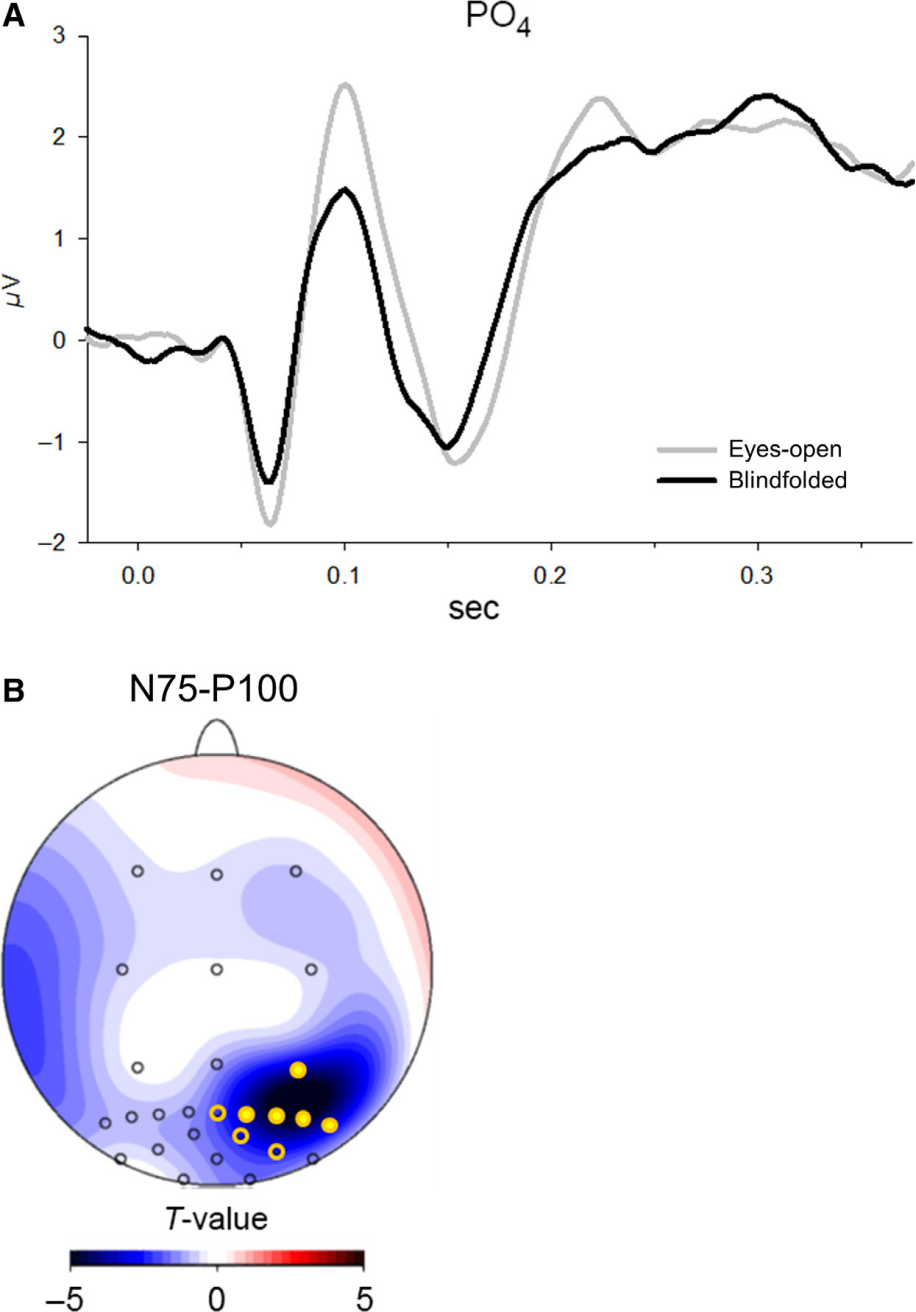
Decrease in the average (*n* = 13) peak‐to‐peak amplitudes of VEPs after blindfolding. (A) Averaged VEP over electrode PO4. *X*‐axis indicates time in s with 0 representing the stimulus onset (pattern reversal), *Y*‐axis indicates amplitude in *μ*V. (B) Differences in the peak‐to‐peak‐amplitude between the N75 and the P100 amplitude are indicated by statistical *t*‐values with negative values indicating lower peak‐to‐peak amplitude for the blindfolded than the eyes‐open condition. Significant differences at specific electrode locations are indicated by filled yellow circles (*P* < 0.01) and unfilled yellow circles (*P* < 0.05). VEP, visual evoked potentials.

There was a distinct effect of blindfolding on VEPs. Analyses of peak‐to‐peak amplitudes revealed a significantly decreased N75‐P100 amplitude after blindfolding as compared to the eyes‐open condition (*F*_(1,12) _= 4.44; *P* = 0.05, Fig. 3A). Although the effect seemed to be most pronounced in right parieto‐occipital electrodes there was no significant interaction for *Blindfolding* × *Topography* (*F*_(26,312) _= 1.765; *P* = 0.174, Fig. 3B). No other significant changes were revealed after blindfolding on amplitude or latency measures of VEPs. For exploratory purposes, we calculated correlations between individual difference (blindfolded minus eyes‐open condition) values of the N75‐P100 VEP amplitude and of SWA during prior sleep. However, none of these correlations revealed to be significant (*r* < 0.42, *P* > 0.155).

### Hormones, subjective measures and attention

Cortisol and melatonin levels were comparable between the blindfolding and eyes‐open condition throughout the recording period (*P* > 0.12). Nocturnal maxima in melatonin concentrations were 58.3 ± 7.8 pg/mL and 62.2 ± 10.5 pg/mL for the blindfolding and eyes‐open conditions, respectively (*P* > 0.322). There were also no differences in subjective tiredness (means ± SEM at beginning of the experiment and before bedtime: 2.53 ± 0.13 vs. 2.47 ± 0.31 and 2.87 ± 0.19 vs. 2.80 ± 0.17; *P* > 0.8) or mood (10.80 ± 0.31 vs. 10.47 ± 0.47; *P* > 0.24 and 11.40 ± 0.24 vs. 11.47 ± 0.26; *P* > 0.83).

## Discussion

The present results show that suppressing visual input through blindfolding the eyes during wakefulness decreases EEG slow wave activity (SWA) during the first 20 min of subsequent NonREM sleep. Contrary to our expectation, the effect did not show a clear maximum over the visual cortex, but involved also other cortical areas, particularly right frontal and central and left parietal cortical areas. VEP responses in the morning after sleep revealed a significantly reduced N75‐P100 amplitude after blindfolding. Interestingly, total time in SWS increased after blindfolding. The global decrease in SWA following decreasing exogenous visual input corroborates the view that information encoding during waking is closely linked to increased synchrony of cortical neuronal network activity during ensuing NonREM sleep, as it has been posed by the synaptic homeostasis hypothesis (Tononi and Cirelli 2003, 2014).

SWA has been considered an electrophysiological correlate of global synaptic potentiation and connectivity in cortical networks. This hypothesis is supported not only by a number of computational modeling studies (Esser et al. 2007; Riedner et al. 2007; Olcese et al. 2010), but also by experimental data showing correlations between SWA and changes in cortical synaptic density across the lifespan. SWA reaches a peak in adolescence and declines thereafter, and the same dynamic across the lifespan is observed for global cortical synaptic density (Kurth et al. 2010; Buchmann et al. 2011; Ringli and Huber 2011; Feinberg and Campbell 2013; Huber and Born 2014). Concurrently, SWA is homeostaticly regulated, globally increasing over the cortex after extended periods of wakefulness and returning to baseline levels across sleep (Borbély and Achermann 2000). The homeostatic regulation has also been considered a consequence of underlying changes in synaptic potentiation and connectivity in cortical networks, increasing during wake due to enhanced encoding of information and decreasing across periods of NonREM sleep, due to synaptic renormalizing effects of SWA and, specifically, the slow oscillation (Tononi and Cirelli 2003; Vyazovskiy et al. 2011). Thus, increasing encoding of information in sensorimotor systems during daytime wakefulness is followed by increases in sleep SWA and vice versa, decreasing encoding of such information decreases ensuing SWA (Huber et al. 2004, 2006). Moreover, increases in SWA after prolonged wake periods coincide with increases in the expression of markers of synaptic potentiation in cortical tissues (Vyazovskiy et al. 2008; Dash et al. 2012). Against this backdrop, this study is the first to reveal a decrease in SWA after suppression of visual input during prior wakefulness in humans. Together with similar observations in animals (Miyamoto et al. 2003; Lesku et al. 2011), it thereby provides evidence that the link between wake encoding and sleep SWA, beyond sensorimotor systems, might also hold for the visual system.

In fact, also the temporal dynamics of the decrease in SWA after blindfolding well fits with previous findings regarding the sensorimotor system (Huber et al. 2004, 2006). Like in those studies we found the effect to be restricted to the first nocturnal NonREM sleep epochs, specifically to the first 20 min of NonREM sleep. There was no change in SWA and related oscillatory activity after 60 min of NonREM sleep. This focus of the effect on the first 20 min of NonREM sleep is in line with the general temporal dynamics of SWA showing its maximum in this initial period of sleep (Aeschbach and Borbely 1993; Andrillon et al. 2011), and thus indeed speaks for an immediate impact of the blindfolding manipulation on SWA.

However, contrasting with those previous studies (Huber et al. 2004, 2006) which revealed local increases and decreases in SWA over respective sensorimotor cortical areas presumably involved in encoding of the task information during prior wakefulness, here the decreasing effects of blindfolding on SWA were distinctly more global in nature, not only involving occipital visual cortex areas but also frontal, central and parietal cortical areas. For the 1–4 Hz delta range of the SWA as well as for discrete slow oscillations statistically significant maximum decreases were obtained that, rather than on occipital recording sites, concentrated over right frontal and central cortex areas. Sleep SWA can be regulated locally (Murphy et al. 2011; Pugin et al. 2015), and this regulation has been related to plastic changes in circumscribed brain regions which have been affected by learning and encoding of specific information during prior wakefulness (Huber et al. 2004, 2006; Hung et al. 2013). Also, signs of decreased SWA that were restricted to visual areas were seen after monocular deprivation in pigeons (Lesku et al. 2011) and after dark‐rearing in mice and cats (Miyamoto et al. 2003). Against this backdrop, the rather global topography of the decrease in SWA after blindfolding, at a first glance, might be unexpected, all the more so since our experimental procedure aimed at keeping the total information presented during the experimental wake period comparable between conditions (i.e., subjects during blindfolding listened to the same story which they read during the eyes‐open condition etc.).

However, carefully considering the constraints of our blindfolding procedure reveals that a more global cortical decrease in SWA activity is in fact not an unexpected outcome. Reading a book, that is, one of the activities of the participants in the eyes‐open condition does not only involve visual cortical areas, but also recruits many more areas implicated in the higher‐order analysis of visual inputs. Thus, changes in synapse formation and markers of synaptic potentiation following blindfolding are probably not restricted to visual cortex areas but also to other cortical areas (e.g., Bengoetxea et al. 2012). An essential contributing factor probably is the high connectivity of primary and secondary visual cortex areas with other cortical areas; indeed ~25% of the human cortex is involved in processing visual information (Van Essen and Drury 1997; Tootell et al. 2003). Thereby, suppressing exogenous visual input would be expected to affect many other cortical sites in addition to the visual cortex, and could even explain that effects of blindfolding were stronger at these extrastriate cortical sites. Thus, synaptic changes after blindfolding might be more pronounced in attention‐related areas controlling input to visual cortex rather than in visual cortex itself, in line with the fact that SWA displays its maximum over prefrontal cortex rather than over any specific sensory or motor area (Kurth et al. 2012). However, if so, it could also be argued that blindfolding should have increased SWA as subjects had to orient without visual information and redirect attention to auditory and somatosensory inputs over more than 10 h. This was an entirely novel experience to all of the subjects, presumably going likewise along with widespread synaptic plastic changes in different cortical areas. Thus, the blindfolding procedure adopted in this study to totally suppress visual input, entails clear limitations if compared with previous published studies aiming to suppress somatosensory input from circumscribed body regions (Huber et al. 2004, 2006). Whatever the case, the rather global decrease in SWA and slow oscillations after blindfolding with maximal effect sizes over right prefrontal and parietal rather than occipital regions suggest that processes of synaptic renormalization and reorganization during sleep do not remain restricted to the respective sensory cortical areas.

It could also be argued that, rather than on synaptic connectivity within cortical networks, the effects of blindfolding were primarily mediated via an influence on hypothalamic circadian systems. Light is one of the strongest zeitgebers, and a recent study by Chellappa et al. (2014) demonstrated that a 2‐h exposure in the evening to blue‐enriched light, compared with non blue enriched light, induced a significant increase in SWA during subsequent NonREM sleep. However, these effects were seen only in subjects with a PER (5/5) polymorphism in the clock gene Period3, and it was associated with a modulation of melatonin concentrations. Here, we did not genotype our subjects with regard to Period3, and hourly analyses of melatonin concentrations did not reveal any significant difference between the blindfolding and eyes‐open conditions. Moreover, effects of blue‐enriched light on SWA in the Chellappa et al. (2014) study was restricted to occipital cortical areas and was observable over the whole night, whereas here decreases in SWA after blindfolding were much more widespread and observable only during the first 20 min of NonREM sleep. Consequently, although due to the limited comparability of the two studies a contribution of circadian oscillators to the effects of blindfolding cannot be entirely excluded, such contribution is likely to be minor.

Surprisingly, blindfolding increased the time subjects spent in SWS when taking the entire night into account which contrasts with the decrease in SWA observed in the initial 20 min. This finding is difficult to explain. Exploratory posthoc analyses did not reveal any significant correlation between these phenomena, suggesting that the increase reflects a process unrelated to synaptic renormalization. We suspect that this increase might be related to the blindfolding procedure representing a task that was not only novel but also very demanding and tiresome. Increase in SWS have been observed after tasks that were moderately mentally and physically stressful to the subjects (e.g., Kern et al. 1995). However, the present data indicating that ratings of tiredness as well as concentrations of the stress hormone cortisol before the sleep period were comparable between the blindfolded and eyes‐open condition, do not support this view.

The decrease in VEP N75‐P100 amplitude in the blindfolding condition represents another observation that is unexpected in the framework of the synaptic homeostasis hypothesis (Tononi and Cirelli 2014). VEPs were recorded in the morning after sleep as another indicator of network connectivity and synchronization in visual cortical areas with, increased VEP amplitude considered a reflection of increased connectivity. Assuming that SWA contributes to the homeostatic regulation of cortical synaptic connectivity by re‐normalizing synaptic strength, reduced SWA after blindfolding should have compensated for the globally reduced synaptic potentiation in visual cortex such that in the morning after sleep VEP responses were expected to be comparable for the eyes‐open and blindfolding condition, as it has been observed in a similar study for SEP responses in the morning after a period of arm immobilization on the foregoing day (Huber et al. 2006). However, we caution against overestimating this observation, because VEP measures were introduced here merely as an additional control in the end of the session. In order to avoid any visual stimulation during the blindfolding period before sleep, we did not assess differences in VEPs in the evening before sleep (which should have been much more pronounced than after sleep). In the absence of such reference data, and also considering that sleep inertia might have differently affected VEP responses in the two conditions, any interpretation of the observed changes in VEPs remains tentative.

In sum, decreasing visual input during daytime wakefulness in healthy humans induces a complex pattern of changes in oscillatory EEG activity during subsequent NonREM sleep. This pattern includes an initial decrease in SWA that is consistent with conditions of reduced synaptic potentiation in cortical networks after daytime blindfolding. However, the widespread topography of this decrease, the overall increase in SWS, as well as the divergent temporal dynamics in changes in spindles suggest that the cortical response during NonREM sleep to prior blindfolding comprises more than a renormalization of synaptic connections in local visual networks.

## Conflict of Interest

None of the authors has any conflict of interest.

## Data Accessibility
